# Industrial cryo-EM facility setup and management

**DOI:** 10.1107/S2059798320002223

**Published:** 2020-04-06

**Authors:** Kasim Sader, Rishi Matadeen, Pablo Castro Hartmann, Tor Halsan, Chris Schlichten

**Affiliations:** a Materials and Structural Analysis, Thermo Fisher Scientific, Achtseweg Noord 5, 5651 GG Eindhoven, The Netherlands; b Diamond Light Source, Electron Bio-Imaging Centre for Industry, Oxford, United Kingdom

**Keywords:** cryo-EM, facility setup, facility management, industry

## Abstract

Cryo-electron microscopy (cryo-EM) has rapidly expanded with the introduction of direct electron detectors, improved image-processing software and automated image acquisition. Its recent adoption by industry, particularly in structure-based drug design, creates new requirements in terms of reliability, reproducibility and throughput. In 2016, Thermo Fisher Scientific (then FEI) partnered with the Medical Research Council Laboratory of Molecular Biology, the University of Cambridge Nanoscience Centre and five pharmaceutical companies [Astex Pharmaceuticals, AstraZeneca, GSK, Sosei Heptares and Union Chimique Belge (UCB)] to form the Cambridge Pharmaceutical Cryo-EM Consortium to share the risks of exploring cryo-EM for early-stage drug discovery. The Consortium expanded with a second Themo Scientific Krios Cryo-EM at the University of Cambridge Department of Materials Science and Metallurgy. Several Consortium members have set up in-house facilities, and a full service cryo-EM facility with Krios and Glacios has been created with the Electron Bio-Imaging Centre for Industry (eBIC for Industry) at Diamond Light Source (DLS), UK. This paper will cover the lessons learned during the setting up of these facilities, including two Consortium Krios microscopes and preparation laboratories, several Glacios microscopes at Consortium member sites, and a Krios and Glacios at eBIC for Industry, regarding site evaluation and selection for high-resolution cryo-EM microscopes, the installation process, scheduling, the operation and maintenance of the microscopes and preparation laboratories, and image processing.

## Abstract 2

The setup and operation of an industrial cryo-EM laboratory is described.

## 1. Introduction   

Transmission electron microscopy (TEM) laboratory designs and cryo-EM facility management play a crucial role in the optimal operation of the microscopes and their output (O’Keefe *et al.*, 2004 ▸; Muller *et al.*, 2006 ▸; Martínez, 2014 ▸; Alewijnse *et al.*, 2017 ▸; Clare *et al.*, 2017 ▸; Kandiah *et al.*, 2019 ▸). However, laboratory-design reports are focused largely on materials science and facility management on academic cryo-EM. The present report aims to address laboratory design and facility management both specifically for single-particle cryo-EM and in an industrial context.

The Cambridge Pharmaceutical Cryo-EM Consortium (the Consortium) was conceptualized by Dr Richard Henderson (Medical Research Council Laboratory of Molecular Biology; MRC-LMB), Dr Harren Jhoti (CEO, Astex) and Dr Peter Fruhstorfer (VP, FEI) in 2015. Pharma companies were looking for shared-risk access to cryo-EM and shared learning. Thermo Fisher Scientific (then FEI) was looking to evaluate the value of cryo-EM in the drug-discovery process and to provide industry access to cryo-EM. The MRC-LMB provided knowledge transfer and expertise.

An industrial cryo-EM facility was therefore formed in early 2016 with a Krios (G2) and a sample-preparation laboratory at the University of Cambridge Nanoscience Centre. The facility is funded by the pharma companies and is operated by Thermo Fisher. The Consortium companies share use of the Krios amongst themselves and also with the University of Cambridge (in exchange for hosting them) and the MRC-LMB (in exchange for expertise and guidance on the use of cryo-EM). With the success of the first Krios and growing demand, the Consortium expanded with a second Krios (G3i) located in the University of Cambridge Department of Materials Science and Metallurgy in 2018. The separation of the two microscopes was owing to mutual Thermo Fisher/materials science interest in diffraction techniques and soft materials, available space in the purpose-built Wolfson Electron Microscopy Suite in the Department of Materials Science and Metallurgy (with extremely stable room temperature control via passive water baffle cooling and low vibrations owing to a 35 × 25 × 2 m concrete slab) and space constraints at the Cambridge Nanoscience Centre.

The Consortium focuses on sample vitrification and data acquisition, with vitrification, sample screening and evaluation, high-resolution data collection on Falcon 3, Falcon 4 and K3-Bioquantum, and individual training on any aspects of the workflow. However, data storage, data transfer and image processing are an integral part of the single-particle analysis (SPA) workflow, and to address these aspects the Consortium has established a robust high-performance storage system, an enterprise 10 Gbps network and a high-performance computing server sufficient for high-resolution reconstructions. The optimal operating condition of the microscopes is validated by an internal Thermo Fisher automated apoferritin pipeline. The Consortium setup offers on-the-fly pre-processing (scheduled *RELION*; Zivanov *et al.*, 2018 ▸) and provides advice for the improvement of data collection and close links to the expertise at the MRC-LMB.

Recently, most Consortium members have acquired in-house cryo-EM sample-preparation laboratories and microscopes. eBIC for Industry at Diamond has set up both a Krios G3i with K3-Bioquantum, a Glacios and a vitrification laboratory with a Thermo Scientific Vitrobot Mk IV, and now provides a full SPA workflow execution and consultancy service for grid preparation, sample screening, high-resolution data collection and image processing. The report here provides a summary of our experiences during and our recommendations on setting up and advising on the multiple facilities that we have been and are involved with.

## 2. Cryo-EM laboratory design, installation and setup   

### 2.1. Electron-microscope rooms   

Modern TEMs are precision tools that have stringent requirements for optimal operation. Designing the construction or retrofitting of a room for a TEM is a significant exercise, and requires careful attention to avoid pitfalls. In addition, most of the literature on room design has come from materials science and is primarily concerned with scanning TEM (STEM), which has different requirements to those for bright-field phase-contrast cryo-EM of biological specimens by SPA or tomography. There are four key parameters: temperature stability, vibrations, acoustic noise and electromagnetic fields.

Temperature stability is a critical factor to the overall stability of the optics. Thermo Scientific Titan, including Krios, TEMs have all constant power lenses to keep the dissipated power (and therefore the temperature) constant. The room-temperature change needs to be less than 0.8°C peak to peak over 24 h. In addition to temperature stability, air-flow velocity is also important, with the minimum air flow desirable (Muller *et al.*, 2006 ▸). With changes in temperature there will be beam drift (and likely beam-tilt changes), and a change in the position of the zero-loss peak if using an energy filter. The Krios cube/enclosure creates its own environment, with a temperature variation far smaller than the room itself. All doors should be closed during use, and permission obtained before any alterations are made to the cube itself, which is also an acoustic damping enclosure. We try to keep the Krios cube, and the room doors, closed as much as possible to avoid temperature fluctuations. Glacios TEMs have constant power objective lenses, but all other lenses are not. In addition, the Glacios does not have a full enclosure like the Krios cube, and it is more sensitive to temperature variation (but with a corresponding specification for performance).

Both acoustic noise and floor vibrations can excite resonances in the microscope and cause deviations to the electron beam, which are critical for STEM imaging, as they cause deviations in the position of the electron probe. For bright-field TEM imaging the relationship is not as clear, but movement of the sample holder and sample itself is possible. In any case, meeting the STEM-derived thresholds should guarantee TEM performance.

Hard specifications for acoustics and vibrations are no longer provided. The most important reason is that both are interlinked. The Site Evaluation tool (SE-tool) represents decades of work on the response of Thermo Scientific microscopes to environmental factors, and takes this inherent dependency into account precisely for the system under evaluation. An electron microscope is typically sensitive to acoustics in the low-frequency range, approximately <500 Hz.

In preparing a site, it is highly recommended to incorporate the traditional sound-reducing measures generally known to building architects: carpet, soft walls and damping ceilings. It is even more important to invest in very quiet heating and air-conditioning systems, especially in the low-frequency region.

An electron microscope is sensitive to floor vibrations, but the frequency range is limited to below approximately 10 Hz. Thermo Scientific microscopes have passive air-cushion isolation systems that provide enough passive isolation above 10 Hz for virtually all sites. For the sub-10 Hz region, Thermo Fisher intentionally does not provide separate, hard specifications for the floor. The most important reason is that a floor, with its three individual directions, cannot be seen as independent from other site-related disturbances such as acoustics. The SE-tool automatically takes this inherent dependency into account precisely for the system of interest (as the platforms are tested on a vibration generator). In the event of a sub-10 Hz failure, it is possible to add an active vibration isolation option: iVIS (Integrated Vibration Isolation System). The iVIS is a six-element system added onto the passive isolators, between the isolated world and the fixed frame, with sensors and actuators, that improves isolation significantly on the critical supported resonances of the microscope on the air isolators, actively damping disturbances within the range 1–5 Hz.

The general design guidelines that we recommend for a new building (yet to be constructed) are as follows.

(i) General guidance in previous Thermo Fisher pre-installation manuals was given in the form of vibration criteria (VC) curves for vibrations (Gordon, 1999 ▸). Architects should not adhere to this guidance and the existing, widely known VC curves. These give an incorrect indication of the requirements with respect to the needs of the microscope. Since VC curves are flat (while the microscope resonance curves are not) they overestimate high-frequency regions by a large amount to meet what is required in the sub-10 Hz range, and non-optimal construction choices may be made based on these. Spring-mounted concrete slabs, or slabs with elastic infill, damp higher frequencies, but at the expense of lower frequencies (duplicating the action of the air suspension damping), and while they may help to meet a VC curve they will not optimize performance as they will increase the sub-10 Hz frequencies. Also, VC curves are in different units to the inputs required for the SE-tool (velocity in µm s^−1^ compared with acceleration in µ*g*). Under limited circumstances the two can be interconverted (identical 1/3 octave bins). Consequently, if a site survey is performed by an external engineering/vibration consultant, they will report vibrations in µm s^−1^ which may not be comparable to the SE-tool output (µ*g*), and the input files are difficult to convert. For example, in one case of the design and building of an extension, a very extensive vibration analysis of the intended site was performed by an external vibration/engineering consultant (continuous monitoring for about two days). Video cameras were used to correlate vibration events with events on the site (mostly large vehicles passing over speed bumps). The peak events were ∼100 Hz (a frequency heavily damped by the passive air-cushion system). These peak events corresponded to VC-C and VC-B (orders of magnitude above the VC-E guidance that was given). It was possible to manually input the data into the correct format by using an existing Spicer Consulting survey kit file and editing it in Matlab. These maximum 1 s r.m.s. values for the entire measurement period passed the SE-tool test for a Glacios and Krios. However, the worst site frequencies are often 8–12 Hz, and very generally planning for VC-E/F at 1–10 Hz with VC-C for higher frequencies (validated by the SE-tool) is good building practice (Lynden Spencer-Allen, personal communication). Values for vibrations should be presented as both velocity (for general VC curve comparison) and acceleration (for comparison with the SE-tool output). In terms of building choices, the costs of passive damping versus active damping (*e.g.* iVIS) should be compared.
(ii) The best base for the Krios and Glacios is a solid concrete block founded directly into the ground of the site at grade and not suspended between discrete points.
(iii) Building and/or floor resonances as low as the critical range of our systems, *i.e.* <10 Hz, should definitely be avoided.
(iv) Under the above assumption, a site survey with the Thermo Fisher SE-tool is recommended to be performed on the bare building location, just in the soil or on a solid asphalt road or slab, before the building is erected.

However, while an initial survey of the vibrations on a particular site are important, in the case of an extension/building the surrounding conditions can easily change over the course of building or before a microscope is installed. Active vibration compensation (Preumont, 2011 ▸; Tjepkema, 2012 ▸) can adapt to changes in the surrounding environment and has become very good, driven in part by the requirements of semi-conductor fabrication plants (Fabs) with nanometre-precision processes. A good example given by Preumont of the potential of active compensation (here of an optical device) is the real-time correction of atmospheric disturbances for terrestrial telescopes, allowing improvement of the resolution by an order of magnitude. Tjepkema highlights in a two-sentence summary of his thesis that ‘(a 45 nm) microprocessor can only be made using precision equipment in which a vibration isolator is applied’.

Active compensation for electron microscopes takes the form of antivibration tables that the whole microscope (ideally only the column frame) sits on and actively compensates a wide range of frequencies, and more recently the iVIS from Thermo Fisher, which is built into the frame of the microscope and compensates for frequencies of 1–5 Hz. The iVIS does not require any special preparation, but in the case of the construction of a new building or extension in which it was not known whether an iVIS would be sufficient, one practical strategy has been to create isolated 1 m deep slabs of several square metres in area, set 50 cm (this likely could have been less) below the planned floor level for the microscope base, before the construction of the rest of the building, and then tested with the Thermo Fisher SE-tool. In the case that the slab fails and active vibration compensation is required, the space could be used to inset an active vibration table underneath the column. Generally, the point is to make sensible investments in the building and use technology to make up the difference if required.

Static electromagnetic interference (EMI) fields do not cause microscope performance issues, but changing fields, especially at very low frequencies, can. However, good field-cancellation systems are available (for example from Spicer Consulting), but a number of issues need to considered. One issue is that field-cancellation systems require loops of cables to be installed, so it is advisable to have these installed as a precautionary measure when a room is being built or refurbished. If there is then a problem or a change in the site conditions over time, a field-cancellation system can be easily and quickly installed. While in-cube field-cancellation systems now exist, the smaller diameter of the field-cancellation coils will cause a steeper field gradient, which may mean that only one point in the cube is optimally field-cancelled. The field gradients with cancellation coils in larger rooms will be shallower, so the difference between different points on the column will be reduced. Field cancellation is possible for both AC and DC fields. However, if large DC fields go outside the range of the field-cancellation system the system needs to be reset. In this ‘new’ DC environment, the microscope alignments may be significantly different. In extreme cases passive shielding including eddy current shielding with a good conductor such as thin aluminium sheets may be used (Muller *et al.*, 2006 ▸).

While much emphasis has been placed on humidity control for cryo-EM microscope rooms (O’Keefe *et al.*, 2004 ▸), the Consortium does not have specific humidity control and the humidity in the Krios 1 room was 40–50% when measured in early 2016 (specification <80% RH). Humidity control was possibly a greater concern for microscopes that used side-entry cryo-EM holders, as they needed to be transferred from the liquid nitrogen-cooled loading stations to the microscope compustage, with potential ice-crystal contamination from the ambient humidity being a real possibility. In the autoloader-equipped systems at the Consortium we do not see a major problem. Overall grid-contamination rates in the column will to some extent be influenced by room humidity, but we find other factors such as cryo-cycling schedules have a much greater influence. Also, ice contamination in the column has been dramatically decreased in the Krios G3i with improved anti-contaminators. 1.5 days after splitting the Consortium G3i column for a service intervention an apoferritin data set was collected, with no visible contamination, and reconstructed to 2.5 Å resolution. Much more time and scheduled cryo-cycles would be required on pre-Krios G3i systems. Given improved anti-contaminators, and the large influence of temperature on microscope performance, it may be beneficial to put more resources into temperature control than humidity control. 30–40% RH at 20°C should be achievable with a reasonable cost, but going to 10–20% may become very expensive. The eBIC/eBIC for Industry Krios hall has 30–40% RH at 20°C.

The design of rooms must also meet many other conditions specified in pre-installation manuals. For a first instrument there is a significant amount of work to ensure that a room, and the location of a room, will be suitable for a cryo-TEM installation.

### 2.2. Microscope installation   

Cryo-TEMs are large instruments, and appropriate internal and external delivery routes must be available to the installation rooms for up to 20 large crates. The placement of microscopes often needs to be decided well in advance of the installation for the delivery of services (water, compressed air and chilled water) and depending on whether EMI fields exist in the optimal position given the space. The operation of cryo-TEMs often requires pressurized liquid-nitrogen Dewars, which will require permanent oxygen sensors with alarms both inside/outside the room, as the failure of a 240 litre Dewar will quickly displace all air in most rooms even with high air flow. Tilt alarms should be used out of hours. A 240 l Dewar will last more than a week, which is a practical exchange cycle (optimally two are present, with one kept filled, and are swapped weekly). During the microscope-installation process sulfur hexafluoride (SF_6_) gas is required as an insulating gas for the high-tension tank and gun. As SF_6_ is a potent greenhouse gas it is tightly regulated and may take some time to acquire (including a use/safety audit) in the European Union and other locations. A Krios also has an acoustic enclosure which needs to be fixed to the floor with large bolts (large holes need to be drilled into the floor and bolts set in the holes with a resin). High-quality/density concrete slabs are often steel reinforced. Having the anchor bolts avoid the steel reinforcement is difficult, but necessary, in order to prevent the creation of alternate ground paths from the cube. Therefore, specialist drilling contractors are recommended.

### 2.3. Cryo-EM preparation laboratory setup   

While many laboratories spend significant effort and money to maintain low humidity in their preparation rooms, although recommended, this was not an option for either of the Consortium preparation laboratories. The main effect of high humidity in preparation rooms is that ice crystals form quickly in liquid nitrogen from the water in the air. Ice is deposited on grids (usually as large crystals), and increases the downstream work of selecting holes for data collection. However, the amount of ice deposited is a function of time. By quickly freezing grids with fresh nitrogen and liquid ethane, we do not find the lack of humidity control to be a major issue. However, we obtained several duplicates of many of the sample-preparation tools to (i) allow us to freeze quickly and (ii) exchange fresh tools for those with ice in the case that longer periods of time are required or large numbers of grids are frozen.

The core items that are required for a cryo-EM preparation laboratory are a plunge-freezing device, usually robotic such as the Vitrobot, a glow-discharge unit, a cylinder of ethane gas, preferably with a two-stage regulator, and enhanced air extraction for safety (usually a fume hood).

Like many other laboratories, we find that the best liquid-nitrogen vessels for the final steps are simple steel vacuum thermos flasks. The main precaution that must be taken is that the flask is never sealed, and one way to ensure this is to never use the lids, but to instead use aluminium foil to cover the top.

### 2.4. IT setup   

Direct detector movies create large amounts of data. However, the hardware, both networking and data storage, to deal with these data is standard in terms of high-performance networking and data storage and is not expensive in comparison to the microscope costs. IT systems are also dynamic, constantly changing and/or growing. The Consortium initially started with five-port unmanaged gigabit switches. When these failed owing to sustained continuous data transfer from uncompressed super-resolution K2 images, consumer-grade 10 Gb switches were installed. The failure of several ports on these prompted us to upgrade to enterprise-grade network equipment. The Consortium also started using 4G mobile data and guest wifi and, as standard business broadband was not available, made a large upgrade to a 1 Gbps leased line.

**Figure 1:**
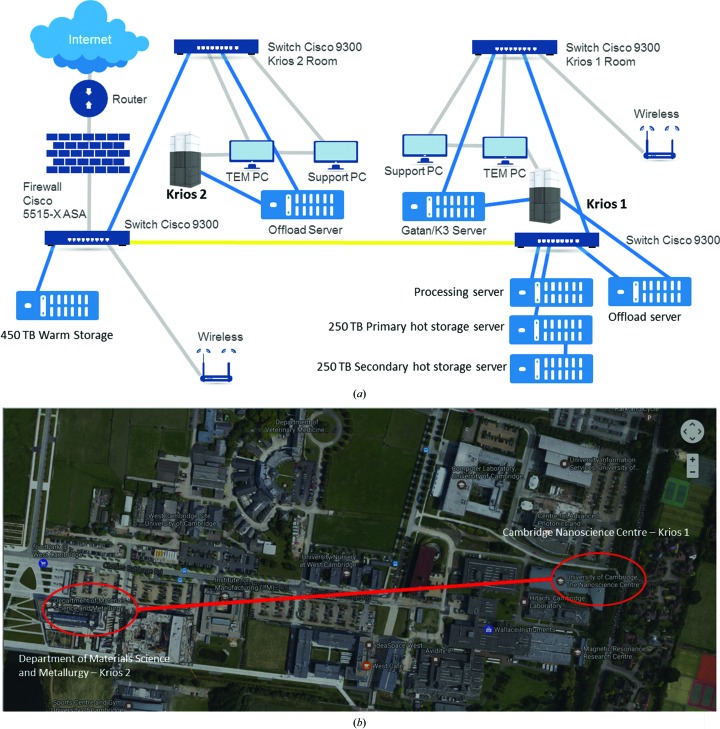
(*a*) Schematic of the Consortium IT setup: the Krios 1 and Krios 2 networks are linked by 3× single-mode dark fibres (yellow line) connected to Cisco 9300 switches with 10 Gb modules in the communications rooms for the Cambridge Nanoscience Centre and Department of Materials Science and Metallurgy, respectively. The 450 TB ‘warm’ storage server, Cisco 5515-X ASA hardware firewall and 1 Gbps leased line router are located in the Materials Science communications room (blue lines, OM3 Multimode Fibre, 10 Gbps; grey lines, 1 Gbps ethernet or fibre). The Materials Science communications room is connected to a second Cisco 9300 switch in the Krios 2 room, which is connected to the TEM PC, Support PC and Falcon Offload server. The Nanoscience Centre communications room houses the processing server and both primary and secondary storage servers, as well as the Falcon Offload server (with a direct OM3 connection to Krios 1). The Nanoscience Centre communications room is connected to a second Cisco 9300 switch in the Krios 1 room, which is then connected to the TEM PC, Support PC and the Gatan/K3 server. (*b*) Physical locations of Krios 1 and Krios 2 on the West Cambridge Site (Google Maps).

The current overall Consortium network is shown in Fig. 1 ▸. Krios 1 and Krios 2 are separated by about 750 m in different buildings, and therefore an enterprise network (Cisco Catalyst 9300 switches) has been critical to bind the two microscopes into a single facility. Access to storage and processing from each site is identical. The main link between the two communications (cooled server) rooms has three dark fibres (single mode) allowing up to 3 × 10 Gb connections. Internal connections are either via OM3 multimode fibres or short Cat6 lengths (also 10 Gb). We make extensive use of remote operation between Krios 1 and Krios 2. We have tried KVM over IP (1 Gbps) sufficient for a full-resolution monitor (https://www.gdsys.de/en/), but we find that the remote-desktop solution *TeamViewer* (https://www.teamviewer.com/) is sufficient for all operations (and the same mechanism is used for offsite remote access). IT infrastructure setups such as this can take some time to establish; fortunately, both sites were connected (Materials Science) or close to (Nanoscience) the University of Cambridge Granta Backbone Network (GBN) with spare dark fibres. Planning was started 6–9 months prior to the Krios 2 installation. Our network is protected by a Cisco 5515-X ASA (adaptive security appliance) hardware firewall.

At the Consortium, we started with 2 × 360 TB 45 Drives XL 60 dedicated storage servers (https://www.45drives.com/wiki/) with Krios 1 and added an additional 600 TB XL 60 server with Krios 2. Especially for the 600 TB server, the cost of the 60 × 10 TB Western Digital Gold hard drives is significantly more than the server itself. These run Linux and a ZFS software RAID filesystem (RAID-Z1, equivalent to RAID 5) and the ZFS pool consists of eight RAID-Z1 VDEVs with seven drives each, with four hot spares set up with an auto-replace function. On-the-fly LZ4 compression was enabled as there is nearly no time penalty for this, and our compression ratio for mostly Falcon 3 counting movies is 1.24×. To give a slight performance increase, atime=off and xattr=xa were set. Our useable space is ∼250 TB and ∼450 TB on the servers, respectively. Storage-grade 3.5′′ SATA drives were used, and each VDEV was created as a physical set of drives to allow troubleshooting in the case of disk failures. As data are spread over many drives, while the read/write speed of an individual SATA drive is ∼100–200 Mbps, together near 10 Gbps is achieved. However, we use these servers as dedicated storage servers, and there is no I/O data transfer for processing. There is a primary storage server (which all data are pushed to/pulled from) and a secondary server to which all of the data from the primary are mirrored periodically. The plan is to keep this two-server setup as ‘hot storage’, with the larger 600 TB server as a more highly compressed ‘warm storage’.

**Figure 2:**
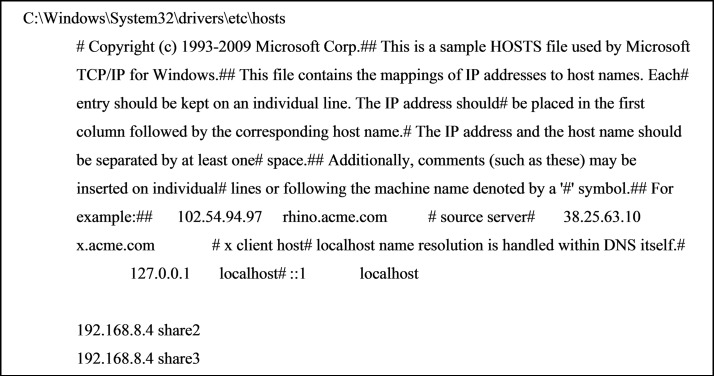
For mounting multiple SMB (Samba) connections with different users on the same SMB server to a Windows host, the different users must be mapped to different host names in the hosts file.

Access to the storage is by username/password-protected Samba shares for each Consortium member on the primary storage system, which is then mounted on various Windows hosts. To allow multiple Samba shares from the same IP address to be mounted on a Windows system, they need to be added as aliases to the ‘hosts’ file (for example C:\Windows\System32\drivers\etc\hosts; Fig. 2 ▸) otherwise they cannot be unmounted and other shares cannot be mounted (the PC needs to be restarted to reset it). These Samba shares are mounted on the Windows machine by ‘Map Network Drive’ and for example \share3\share3 which will prompt the password. 10 Gbps via appropriate Network Interface Cards is achievable on our system (from both the Falcon 3 HP Apollo server and the Gatan K3 PC with RAID array of SSD drives to the primary storage server). Sharing is multiprotocol as the boxes also have NFS shares, which are used for the mirroring of the primary and secondary servers.

Data sets for processing are transferred to a Processing server (Supermicro LGA 2011; quad socket 4× GPU ready; we have 64 cores, 512 GB RAM, 1 Nvidia Titan X, 2× Nvidia 1080 GPUs). We have 12× 2 TB SATA drives in two pools of 12 drives in RAID 10 which perform at the 6 Gbps of the RAID controller. With the MRC-LMB being a consortium member, *RELION* is the main package used, but *EMAN*2 and *IMOD* are also installed for various image-processing tasks (and were especially useful for earlier *RELION* versions when the *RELION* image handler had fewer options and *EPU* movie output required more pre-processing). We have an in-house Thermo Fisher apoferritin pipeline that enables easy benchmarking experiments (also used for Apoferritin Workflow Validations as part of the Thermo Fisher Accelerate Package). We are also experimenting with an HP Server with 20 cores, 386 GB RAM and 2× P40 Tesla GPUs running VMWare.

We have used Amazon EC2 Cloud, which can be especially useful for large peaks of use, and in this case it was set up for use during an image-processing course. Early on we also used a private cloud service (Sabalcore; https://www.sabalcore.com/) which provided a full *RELION* installation and testing service.

#### 2.4.1. Gatan K3 setup   

The Gatan K3 camera was installed at eBIC for Industry in November 2018. *Latitude S* was used as the data-collection software, yielding collection speeds of over 200 images per hour, although recently the K3 has been embedded and is operational through *EPU* at up to 340 images per hour. The K3 is a single electron-counting direct detection camera with a 24 megapixel (5760 × 4092) field of view and a collection speed of 1500 frames per second [note that the maximum frame rate to the PC (server) is 75 frames per second]. At eBIC for Industry we are currently collecting data in counting mode with a dose rate of 15 e^−^ pixels per second and typically a 2 s exposure. The gain reference is taken with the dose rate that we use for data acquisition and is performed prior to data collection.

The current K3 server contains 16 Intel Xenon CPU cores, 256 GB of RAM and a 12 TB array of SSD disk drives running under Windows Server 2012. The server has been placed in a dedicated server room, although to achieve this the five fibre-optic cables from the camera head server need to be replaced and/or patched in the same configuration. An alternative to relocating the server is to use a soundproof cabinet.

## 3. Industrial cryo-EM facility operation and maintenance   

### 3.1. Sample-intake procedure   

The Consortium requires that each sample that is brought on-site is accompanied by a declaration from a designated person from each company that the sample is non-toxic and non-hazardous. Consortium members can either bring samples in person or send them by courier.

### 3.2. Sample preparation   

Initially, many of the Consortium members had little or no experience with the vitrification protocol for grid preparation, and the procedure was performed by Thermo Fisher staff during the microscope sessions. The Consortium members realized that spending time freezing samples while the Krios was unused was not an efficient use of time. As Vitrobot use outside of their booked sessions was only allowed for fully trained users, many Consortium members became fully trained for vitrification very quickly.

**Figure 3:**
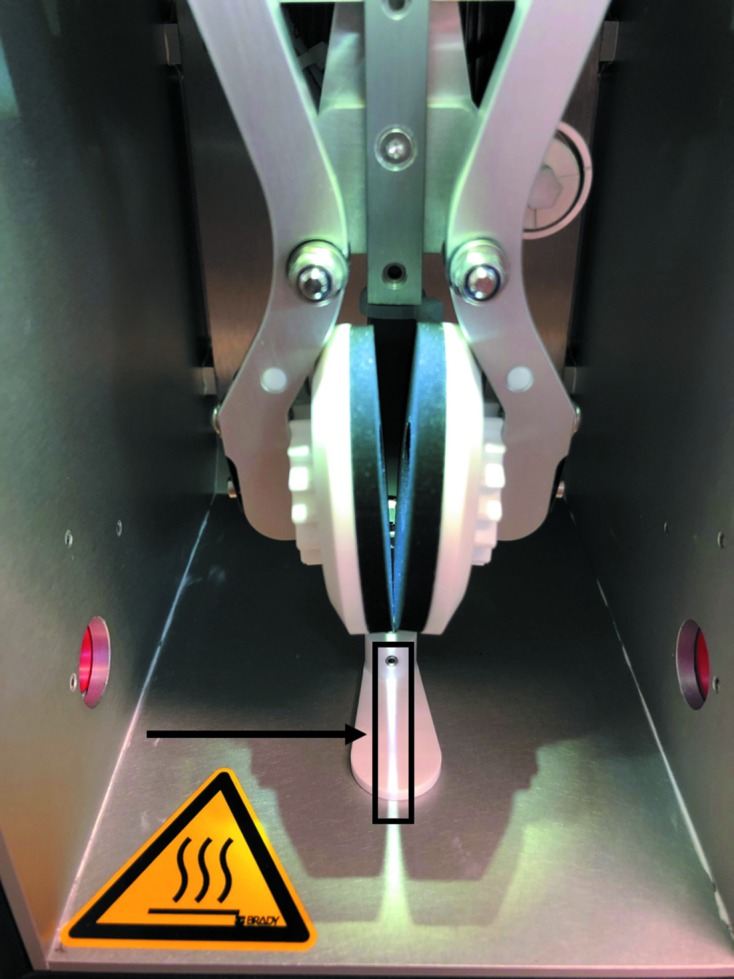
The Vitrobot Mk IV ‘blot-force’ steps are 25 µm offsets of the blot pads. The optimal blot force (and blot-pad separation) to obtain a wedge across the grid containing most ice thicknesses is when the blot pads (without filter paper) are just touching or letting a sliver of light through (indicated by the arrow and rectangle). Fine tuning can then be performed by taking full atlases of grids frozen with ±1 or 2 blot-force steps. Increasing the blot force moves the blot pads closer together and therefore the wedge higher (less thick ice areas). Decreasing the blot force moves the blot pads apart and therefore the wedge down (more thick ice areas).

We find the Vitrobot Mk IV very reproducible and robust in producing grids that contain areas with the desired ice thickness, and this does not depend on user experience (apart from bending or dropping grids). This level of reproducibility does not appear to be the generally accepted experience in many academic laboratories. We have used the same vitrification settings for all of the samples for more than three years on our Vitrobot (Krios 1 preparation laboratory), with thousands of grids frozen so far. The ‘blot force’ (each step is actually a displacement of the horizontal blot-pad position by 25 µm) is the critical parameter that needs to be calibrated/measured once for each Vitrobot. The blot force needs to be set so that the blot pads (without blot paper) are just touching (and letting a sliver of light through them; Fig. 3 ▸; Hervé Rémigy, personal communication). The changes in blot-pad position are only applied after a full blotting cycle (not by ‘resetting’ the blot pads). We apply 2.5 µl of sample with a 2.5 s blot time at 4°C and 100% RH (100% RH is easy to achieve and maintain at 4°C). Also, the first blot after adding the blot paper to the blot pads does not work reproducibly (and requires a full ‘blank’ plunge-freezing cycle with tweezers), possibly because of the displacement of the blot pads while adding the filter paper. To avoid cross-contamination between samples from different companies, we use two blot papers and a ring of Parafilm on each blot pad. This extra thickness required a small adjustment of the blot force compared with a single filter paper. Part of the observation of the reproducibility of the blotting was through the use of full atlases for every grid during screening. On the Falcon 3 a full 4 × 4 tile atlas takes approximately 6 min and provides a great deal of information compared with the traditional practice of manually evaluating a grid on the fluscreen. This allows us to compare every grid that has been screened. We now have two Consortium Vitrobot Mk IVs, and cryo-EM preparation laboratories with Vitrobot Mk IVs at four Consortium members, which are all operated in the same way. With samples of standard viscosity (for example <5% glycerol, sucrose) we get no black or empty grids. We do not find that changing the blot force alters the ice thickness. Changing the blot time may change the ice thickness in the case of much longer blot times, but likely by evaporation from the large air–water interface that is created, which is not desirable. Some Vitrobots show variation in the position of the wedge, but there are always squares with ice thicknesses that need to be analysed by collecting data-acquisition images. The collection of 1000–2000 holes is easily achievable. However, with faster detectors such as the K3, more of the grid squares may need to have the correct thickness, requiring a shallower wedge, face-on blotting or improvements of automated data acquisition to collect data from several grids.

We use Quantifoil Cu R1.2/1.3 300 mesh grids, mostly to remove or at least control another variable. We find significant variation in the hole size even with Cu R1.2/1.3 grids: many boxes have 1.6 or 1.8 µm holes instead of 1.2 µm. We can achieve a 2.2 Å resolution apoferritin reconstruction (1.07 Å pixel) from an overnight data collection with such grids, suggesting that they are not limiting at these resolutions with patch-based movie alignment.

### 3.3. Sample optimization   

One of the most serious problems and current bottlenecks is that biochemical characterizations often do not predict the behaviour of samples in ice. As has been shown by others (Glaeser, 2018 ▸; Chen *et al.*, 2019 ▸; D’Imprima *et al.*, 2019 ▸), the large air–water interface can create major artefacts for many proteins and complexes. We often use graphene oxide grids, initially produced using the procedure of Martin *et al.* (2016 ▸) at the MRC-LMB using their Edwards S150B Sputter Coater, to overcome preferential orientations and aggregation. The procedure used by Martin and coworkers requires Quantifoil grids to be glow-discharged prior to the application of the graphene oxide solution, but many dedicated glow-discharge units with a low maximum current (25 mA) do not appear to work well. One explanation is that the amorphous carbon is not activated enough to allow graphene oxide flakes to stick, although with minimal washing some heavier flakes that have settled may persist. The observation that higher current glow-discharge units (Quorum GloQube; maximum current 50 mA) also work well is consistent with this explanation, and these are now used by the Consortium and several Consortium members. The addition of detergents (for example DDM) to the samples of interest can help to alter their behaviour on the grids. Also, we find that negative stain effectively predicts the behaviour of proteins on thin (2 nm) carbon and graphene oxide. This can be used as a diagnostic test: if negative staining looks good, but the sample is heavily aggregated in holes, it is likely to be an air–water interface problem. 2 nm carbon (if 400 kDa and above), graphene oxide or detergents can then be employed. Recent improvements in graphene-based supports with controlled chemistries (Naydenova *et al.*, 2019 ▸) could be a great addition.

### 3.4. Sample storage   

Each Consortium member has their own liquid-nitrogen storage Dewar that is kept in the Krios rooms, Taylor Wharton (now Worthington) HC20s for Krios 1 and HC35s for Krios 2 (to allow storage pucks), both of which are lockable with padlocks. The Consortium staff are responsible for keeping the Dewars filled, and Consortium members are responsible for the organization/tracking of samples within their Dewars.

### 3.5. Allocation and scheduling   

The Consortium has five company members, initially with an equal allocation of time during weekdays on Krios 1. The MRC-LMB and University of Cambridge share the weekend days. Initially, we scheduled time in blocks of one day, but very early on we realized that having a set day/week for each company was not sufficiently flexible to cope with changing demands over time. So, we changed the policy to allow the booking of single-day sessions up to the allocated total (38 days per year per Consortium member, 9.5 days per quarter; 266 days in total), but averaged over a quarter (so that during a particular week or month one company could use much more than another, but over a quarter it had to be roughly equal). After the first couple of quarters we added non­bookable ‘maintenance’ days to the calendar so that if a particular session needed to be cancelled, it could be rescheduled in the near future. We also have allowed longer sessions (of up to three days) to be booked, as the Krios are the only microscopes at the Consortium, so both screening and data collection are usually performed and the continuity of a longer session is beneficial. We analyse the usage by the Consortium members on a quarterly basis and distribute days remaining in the schedule to those who have used the fewest days over the quarter or over the year as a whole. On Krios 1, which is a G2 Krios, we have discouraged sessions of longer than three days owing to concerns over potential contamination. Apart from this, the quality of samples usually increases with multiple sessions, so five one-day sessions on a project are usually more efficient at collecting high-quality data than one five-day session. With Krios 2, which is a Krios G3i (with improved anti-contaminators) and the improved contamination rates (see Section 3.6.2) on Krios 1 we have allowed much longer sessions (seven days, although usually with multiple grids).

Sessions during which a problem occurs that cannot be quickly fixed (2–3 h), or results in the inability to collect a data set overnight, are deemed to be a ‘down’ day and replacement days are given (from the maintenance days). We have also scheduled some facility ‘development’ days (see Section 3.10).

### 3.6. Microscope operation   

A major difference between the Consortium and other multi-user facilities is that access to the microscope rooms and data is much more tightly controlled. Consortium members are only allowed to be in the microscope rooms during their own sessions, with the permission of the other Consortium members using the microscopes or accompanied by a Thermo Fisher staff member (required to retrieve stored samples from one location to the other). Access to the Vitrobots comes with each Krios session, but Consortium members have been very flexible in sharing access to the Vitrobots outside microscope sessions to allow the efficient use of microscope time.

Both the Consortium and eBIC for Industry conduct a combination of screening and data collection on Krios microscopes, with eBIC for Industry moving to offer screening on a Glacios cryo-TEMs, and several Pharma Consortium members acquiring in-house Glacios. There are a range of user levels, from fully independent to only overseeing the data collection. Users normally come in person to the Consortium, although a full mail-in service is possible for both the solution to be vitrified and prepared grids, with a mixture at eBIC for Industry. We find industrial scientists very disciplined with respect to standard operating procedures. At the Consortium, extensive remote operation is conducted, both of Krios 1 from Krios 2 and of Krios 2 from Krios 1, and externally. We remotely operate the Krios during the ten-day University of Cambridge Christmas/New Year closure period, while there is no physical access to the Nanoscience or Materials Science Buildings, as 20 days of Krios time has a large value. *Teamviewer* has facilitated access via a 4G data connection for over a year (VNC was not useable), but the 1 Gbps leased line acquired provides real-time remote access via *Teamviewer* over which Ronchigram phase-plate alignment is possible. It is also possible to use full hand panels via a *TeamViewer* VPN with port forwarding via the support PC. We have recently started to allow remote operation of the Consortium Krios via *TeamViewer* during core hours (9 a.m. to 5 p.m.) by experienced users and out of hours by expert users. Currently, Consortium user remote operation has been without hand panels, but this may change depending on requests as our remote operation matures.

For the Falcon 3 we collect data sets with a pixel size of 1 or 0.65 Å, a dose rate of 0.5 e per pixel per second, with 60 s exposures and 75 fractions, and one image per hole, giving around 25 images per hour. We observe that this is sufficient for routine 2.5 Å resolution reconstructions for a range of optimized samples. For the Falcon 4 with 1.5 e per pixel per second, we achieve about 60 images per hour with one image per hole and 150 images per hour with aberration-free image shift (AFIS) with a 6 µm cluster size and R1.2/1.3 grids. AFIS is accessed in *EPU* via the ‘Faster Acquisition’ option (with the other option being ‘Accurate Hole Centering’). This mode allows the selection/deselection of arbitrary holes on a grid square as well as the application of a beam-tilt compensation for off-axial coma for beam/image-shifted acquisitions. We are exploring different dose rates, and for large samples that do not need as much low-frequency detector quantum efficiency (DQE), higher dose rates such as 6 e per pixel per second may be appropriate, as the final result is more dependent on the Nyquist DQE and the image rate is also faster at 225 images per hour (which is also why integrating Falcon 2 and Falcon 3 was also sufficient and fast). We achieve around 250 images per hour for the K3 with 12 e per pixel per second collected in super-resolution non-gain-corrected compressed TIF format with AFIS. We advise the use of *RELION*-3.1 optics groups coma refinement on the AFIS clusters when at 2–2.5 Å resolution, as even 0.2 mrad of coma may degrade data at these resolutions. We are planning on creating a fringe-free imaging alignment in the near future to make better use of the optimal ice-thickness areas either with multiple images per hole or smaller holes. However, given that our much lower image rates for Falcon 3 resulted in atomic resolution reconstruction sufficient for all ligand sizes tested, we have encountered different limitations with faster detectors. We need to be able to collect data sets from multiple grids per day, and the fast accurate selection of good-quality holes needs to be made faster for this to occur. We expect these features to be incorporated into *EPU* in the near future. Where features were not available in *EPU*, we have explored third-party commercially supported data-acquisition programs such as Gatan’s *Latitude S* software for K3 data acquisition before embedding the K3 for *EPU*.

#### 3.6.1. Microscope performance/quality checks   

Our main overall performance monitor is the routine determination of high-resolution 3D reconstructions (usually 2.5–3 Å) by Consortium members performing ligand screening. We find 3D reconstructions to be very sensitive to the microscope conditions. When a question arises, we have quality-controlled aliquots of apoferritin (Prospec Bio PRO-650, recombinant human apoferritin light chain, 1 mg ml^−1^) stored in liquid nitrogen. The 1 mg ml^−1^ concentration requires the use of either graphene oxide or 2 nm carbon grids, as not enough particles go into an ice layer. The Thermo Fisher apoferritin pipeline uses standard *RELION* procedures with scaled templates for particle picking based on magnification and particle selection using *RELION* particle-quality metrics, followed by direct 3D refinement and post-processing. The Thermo Fisher apoferritin pipeline is also used for the ‘Workflow Validation’, which is a commercially available option, although a different apoferritin sample is used at a higher concentration so that graphene oxide/2 nm carbon grids are not required. The reconstruction of apoferritin appears to be much more sensitive than the standard information limit test (using Au cross-grating) and Thon ring tests (using Pt/Ir). Both of these tests are not carried out under the same conditions used for data collection (*i.e.* movies in *EPU*) and the same cycles of illumination changes. Better and more uniform strongly scattering amorphous samples could be a route to more quantitative Thon-ring measurements. However, methods have recently been developed to measure the beam tilt (and higher order aberrations) in images by the comparison of projections of particle images with the real structure in *RELION* (Zivanov *et al.*, 2018 ▸) and this information cannot be extracted from single images of amorphous samples.

#### 3.6.2. Alignments   

We find that the core microscope alignments are extremely stable, and changes are usually owing to environmental factors such as changes in the room temperature or user misalignment. Therefore, we only allow a very limited number of alignments to be performed: beam centering, astigmatism, Zemlin beam-tilt tableau determination and correction of coma with the objective aperture removed, followed by insertion and centering of the objective aperture in diffraction mode with the beam over a scattering material (usually Quantifoil carbon) and astigmatism again (contamination on the objective aperture leads to small changes in astigmatism).

The X-FEG emission is very stable, and therefore if the dose rate on a detector has not changed for given illumination conditions (for Krios 1 a setting of Gun Lens 5, spot size 10, beam diameter ∼1.38 µm gave a dose rate of ∼0.5 e per pixel per second at 75k/1.07 Å per pixel on our Falcon 3 for over a year) there is no need for alignment of the gun. Pivot points are probably the most often misaligned direct alignments, as they are only valid at a specific objective lens excitation. In addition, if the beam is not shifting during the beam tilt required for autofocusing, there is no need to adjust the beam-tilt pivot points. On the other hand, beam-shift pivot points are important when performing beam-shifting data collection to increase the data-collection speed with fast detectors such as the Falcon 4/K3. While the off-axial coma caused by moving to the edge of an R2/2 hole may not be significant at 2 Å resolution (for multiple images per hole), this relies on there not being additional beam tilt caused by the beam shift (Cheng *et al.*, 2018 ▸). Therefore, the beam-shift pivot points must be well aligned. The phase-plate beam-shift pivot-point alignment is the most accurate as it uses a real feature in the back focal plane (the carbon of the phase plate). Grouped beam-tilt refinement in *RELION*-3 should also correct for this. We do observe that tuning of the condenser deflectors (condenser zoom) does need adjusting occasionally to optimize C2–C3 alignment, but we only perform this if there is a functional problem with a large beam (hole finding does not work correctly).

#### 3.6.3. Microscope maintenance: cryo-cycles   

**Figure 4:**
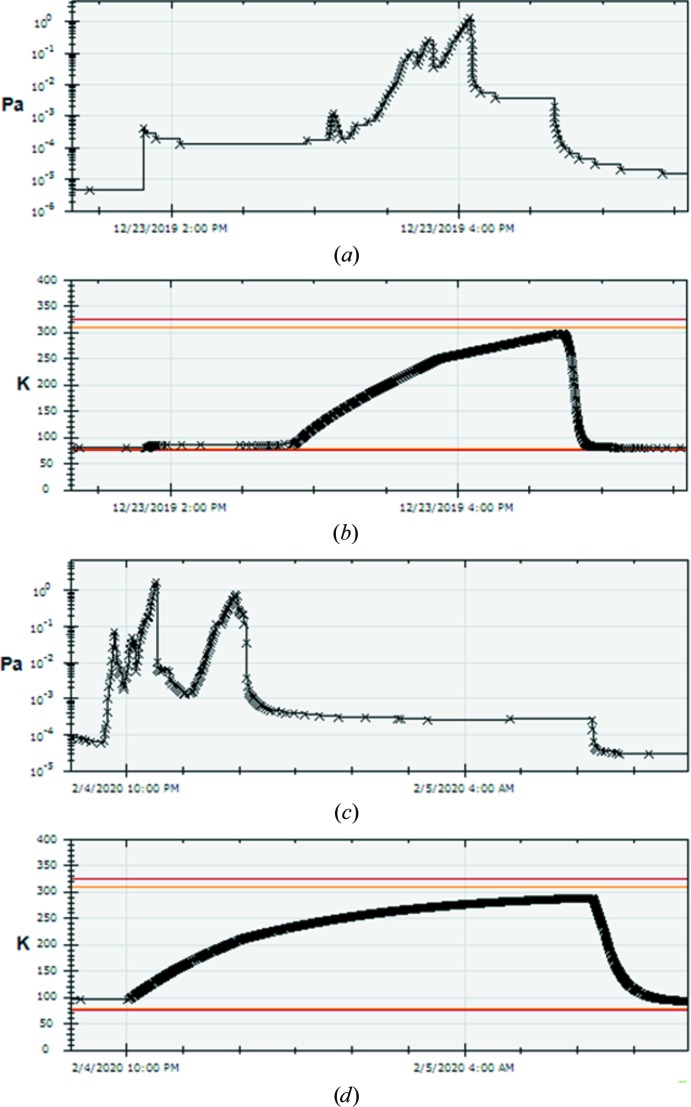
Consortium cryo-cycling procedures. The health-monitoring data from a typical column (*a*, *b*) and autoloader (*c*, *d*) cryo-cycle with vacuum (*a*, *c*) measured by Penning Pirani vacuum gauges (PPcl and PPal) and the corresponding temperatures (*b*) of the sample ‘holder’ and (*d*) the autoloader ‘docker’ show that shorter cryo-cycles of 2.5 h for the column and 4.5 h for the autoloader are sufficient.

Our cryo-cycling schedules are probably the greatest deviation from others in the field. In a 2017 facility best-practice paper (Alewijnse *et al.*, 2017 ▸) practices ranged from a 48 h cryo-cycle every 3–4 weeks to a 24 h cryo-cycle every week. Recent service recommendations suggest a decreased duration of 8 h weekly, although recent software changes enforce a minimum of 12 h. We do 4.5 h for the autoloader (weekly if possible) and 2.5 h for the column (very infrequently, once per quarter). One definition of a full cryo-cycle is that all components have warmed up to room temperature. The column cryobox/holder parts warm to room temperature in ∼2.5 h (Fig. 4 ▸ *b*), and the peaks in water vapour can be followed in health monitoring to ensure that all ice has sublimated (Fig. 4 ▸ *a*). For the column there is no reason to cryo-cycle longer than this, as the cryo-cycle switches the ion-getter pump (IGP), which is a very good pump but not for water, to a turbomolecular pump (TMP; a much worse pump that effectively pumps water vapour). Keeping the column on a TMP for longer actually degrades the vacuum condition of the column. The best practice is to switch to IGPs and cool down as soon as possible (as the cold cryo-box also acts as a vacuum pump). For nearly two years we were able to observe ice growth (‘leopardskin’) on parts of grids after two days. By decreasing our column cryo-cycle length and frequency, the coverage of contamination dramatically decreased and is only visible after much longer periods (seven days).

For the autoloader, which is always pumped by a TMP only, longer cryo-cycles do not degrade the vacuum, but they are not necessary. Plan 3 autoloaders, which most in the field are, have very low ice-contamination rates on grids as the cryo shielding is much better than in the column. However, it is important to avoid ice buildup for proper mechanical/optical sensor function. For the autoloader, while it takes 8 h to fully warm up (Fig. 4 ▸ *d*), if the vacuum is followed in health monitoring all of the water peaks shown by the autoloader vacuum monitor have been removed after ∼4.5 h, as the ice sublimates far below 0°C in a good vacuum (Fig. 4 ▸ *c*).

Since 4.5 h is a relatively short time (similar to selecting holes on some grids for data collection), we have moved away from blocking time for cryo-cycles. We run autoloader cryo-cycles if a sample is not suitable for data collection and the time would otherwise go unused. We have also started performing autoloader cryo-cycles with grids on the column, so that we only need to interrupt an *EPU* run temporarily: stop *EPU*, perform a 4.5 h autoloader cryo-cycle, start cooling the autoloader (0.5 h), turn the TMP to auto-off (the vacuum is good already) and start *EPU* again. This has resulted in a massive increase in useful microscope operation time and allowed us to achieve the use of 355 out of 365 days in 2018 on Krios 1.

As Thermo Fisher staff we have access to more controls/diagnostics on the Krios, often allowing us to diagnose problems and streamline the arrangement of an engineer to fix larger problems, which has also contributed to our uptime.

### 3.7. Data transfer   

At the Consortium, we make extensive use of the Windows program *Robocopy* (https://en.wikipedia.org/wiki/Robocopy), which is part of all Windows installations that we have tested, including Windows 7, Windows 10 and Windows 2012 Server. It is similar to Linux *rsync*, with many options for monitoring folders and transferring or moving files/synchronizing folders periodically with a set time. Specifying paths is easy as they are the Windows-mounted paths and have the same permissions as the current user has (during early trials this was not always the case with external synchronization programs). Some useful flags are /S, which includes all subdirectories, and /MOT, which monitors on time. Our usual transfer command is robocopy /S /MOT:5 <source directory> <target directory>.

Our microscope PCs are directly connected to our local network (not the internet) via Cisco Catalyst 9300 switches. This was required before the Falcon 3 was installed with its HP Apollo server (Offload server) as all data were transferred via the microscope PC. We usually still save all *EPU* data to our primary storage. This splits up the normal *EPU* files (with integrated images) and the movies (written to the HP Apollo server from the Falcon 3 CMTS). Some users prefer to have all files including movies in the same directories. An easy way to perform this is to specify the HP Apollo server as the location for *EPU* (*EPU* will then write its files to the HP Apollo server via the microscope PC, and the Falcon 3 CMTS will write the movies to the same location).

After an *EPU* run has started, the *EPU* session directory is created on the Falcon storage server, and we then set up *Robocopy* to synchronize with the date of collection on the particular consortium member’s Samba share. For the K3, we need to have *Robocopy* move, not copy, the files to the primary storage and leave the directory structure intact (possible via /MOV), as the 10 TB SSD RAID array is only sufficient for 1–2 data sets.

We then mount external hard disks either via a multi-port USB hub, or docking stations for internal drives, and start *Robocopy* to synchronize the collecting data with the hard disk. We also are making use of our 1 Gbps network to copy data on the fly to Amazon S3 using Cloudberry Backup Ultimate, which checks for new files every hour and uploads them directly, with other Consortium members using the open-source program *Syncthing* (https://en.wikipedia.org/wiki/Syncthing) and azcopy to Microsoft Azure.

While we have built a 10 Gb network, this was with future-proofing in mind, and the upgrade was largely for enterprise network hardware rather than for speed. The data rates from both the Falcon 3 and K3 (non-gain-referenced, super-resolution, compressed TIFs) are in the range of 250 Mbps, which is easily accommodated via a 1 Gbps network as long as data are moved on the fly. However, with AFIS/FFI Falcon 4 data collection, we expect our data-collection rates to exceed 1 Gbps.

### 3.8. On-the-fly processing   

**Figure 5:**
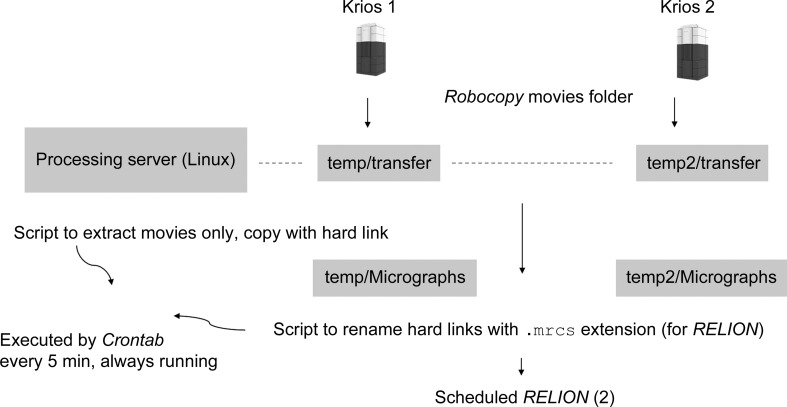
The pharma Consortium on-the-fly setup consists of pushing data on the fly with *Robocopy* to the processing server (to an SMB-mounted folder) and simple bash scripts to extract movies (using globstar) and hard link/rename the files to .mrcs (not required for *RELION*-3). Scheduled *RELION* is then performed, usually for motion correction and CTF determination to ensure data quality, and also enables a fast start to 2D classification if desired. On-the-fly pipelines are a rapidly developing area, but in most cases some reformatting/transferring/data extraction is needed.

There are many packages that can be used for pre-processing (and processing), including *Scipion* (Gómez-Blanco *et al.*, 2018 ▸), *Focus* (Biyani *et al.*, 2017 ▸), *cryoSPARC* (Punjani *et al.*, 2017 ▸) and scheduled *RELION* (Fernandez-Leiro & Scheres, 2017 ▸). However, the setup and maintenance of many diverse packages is time-consuming and costly for industry. Therefore, at the Consortium we have focused on using scheduled *RELION* for on-the-fly pre-processing (Fig. 5 ▸).

A couple of bash scripts are required, but they have been made very simple by setting the bash-shell options (shopt -s) to use wildcards (globstar): shopt -s globstar. Therefore, the command cp -r /test/**/*Fractions.mrc recursively extracts all of the movies from a given directory. We hard link files (cp --l) to the second directory ‘Micrographs’ rather than copying them (duplication wastes space) or moving them (which would create problems with *Robocopy* on-the-fly synchronization).

We have recently upgraded to *RELION*-3, and therefore the final step of renaming with .mrcs extensions is no longer required. We perform movie alignment, now with *RELION* *MotionCor*, and CTF determination. We usually use *Gctf* (Zhang, 2016 ▸), but we may move to *CTFFIND*4 (Rohou & Grigorieff, 2015 ▸) to free up our GPUs for 2D/3D classification/refinement processes, and with 64 physical cores (specified before *RELION* GPU acceleration was released) we have more than enough to keep up with any expected image rates for one Krios, and a second server is being investigated to enable on-the-fly pre-processing with both Krios. With *RELION*-2.1 we were missing an updated graphical display of the results of motion correction and CTF determination. This is now possible with *RELION*-3 by displaying the PDF of results after CTF correction and leaving it open in an X window; it is automatically updated after each round of on-the-fly motion correction and CTF determination (usually 10 min cycles). The Relion_it.py script (Zivanov *et al.*, 2018 ▸) automates the scheduled steps, and we are also exploring this.

### 3.9. Shared learning   

A unique aspect of the Consortium is the shared learning opportunity. The consortium has created a shared learning environment for the participating pharma companies. Since most companies were new to cryo-EM technology, the Consortium enabled them to learn, develop and share best practices in a pre-competitive environment. The Consortium agreements are set up to allow knowledge exchange between companies, while protecting their own IP. Monthly Consortium meetings with a large technical component are organized with both internal and external speakers on a wide range of cryo-EM topics.

As journal articles usually only publish successful protocols, they offer limited guidance in quickly learning from failed experiments. In contrast to academic groups, pharmaceutical companies do not have a knowledge base and years of experience to fall back on. The expertise of MRC-LMB researchers, Thermo Fisher application specialists and the mutual interaction of each company fulfil the place of an academic research group for new PhDs and postdocs.

As the technology is quickly changing, much knowledge is not available in textbooks or mature training offers, and a shared learning environment facilitates the early adoption of these changes.

### 3.10. Research and development projects   

We actively participate in methods-development projects that are directly useful for facility users, including fringe-free imaging, standard test samples (apoferritin), K3 testing and implementation and new techniques in collaboration with our hosts (STEM: dark field/iDPC), scanned diffraction/scanned precession diffraction, microED/diffraction-rotation tomography, and cryo-EM of nanoparticles and soft materials.

## 4. Summary   

In terms of facility operation, vitrification with the Vitrobot Mk IV is very reproducible. Krios alignments change very little over time with stable temperature, and the only routine alignments that are performed are beam shift, objective aperture centering, and automated coma and astigmatism correction. A significant investment in IT expertise is required to store and transfer the large volumes of data produced and to facilitate timely and efficient image processing. Some on-the-fly pre-processing is performed, which is useful for monitoring changes in instrument performance and quickly starting 2D analysis or 3D refinement. With changes to cryo-cycling procedures, we achieved the use of 355 out of 365 days for the Consortium Krios 1 in 2018. These points are generally applicable to both industrial and academic facilities.

There are also several industry-specific lessons that have been learned. Industry users expect access control to their samples, to their data and to the microscope rooms/control workstations during their sessions. The windows for creating impact on drug-discovery projects for structural biology and cryo-EM are relatively small, and therefore industry values robust, reliable and predictable operation as much as absolute performance. The key factor of contribution to drug-discovery projects means that the most significant results may not be published, and therefore journal papers are not a good measure of success for industry. We were already able to achieve resolutions at which the binding of most ligands can be determined (∼2.5 Å) overnight with the previous generation of detectors (Falcon 3), and therefore industry is now pushing for automation of data collection from multiple grids to make the best use of the current developments of faster detectors (Falcon 4, K3), AFIS and FFI (Saur *et al.*, 2019 ▸).

Many aspects of the Consortium have been important for its success. The University of Cambridge has made available two rooms with specifications exceeding those required for a Krios, allowing the installations to proceed immediately after the contracts were finalized. The MRC-LMB has provided expert guidance on setting up and running a cryo-EM laboratory and on all aspects of cryo-EM theory and practice. The companies share risk by pooling their resources together. A key distinguishing feature of the Consortium is the willingness of the company members to share the information that they have been learning between them, and we have facilitated this through a shared learning monthly meeting in which there is usually a significant technical presentation from external and internal speakers. The expansion of the Consortium with Krios 2, on-site company screening microscopes and the creation of an industrial cryo-EM service centre, eBIC for Industry, at DLS, UK demonstrates the success and optimism for the future of industrial cryo-EM.
